# Adolescent alcohol consumption alters sex-specific behaviors associated with prefrontal functional connectivity in mice

**DOI:** 10.1101/2025.06.05.658112

**Published:** 2025-06-08

**Authors:** Laurel R. Seemiller, Hayreddin Said Ünsal, Yiyan Xie, Md Shakhawat Hossain, Kaleah Tuttle, Bryce Johnson-Dendy, Emily P. McDonald, Patrick J. Drew, Nanyin Zhang, Nicole A. Crowley

**Affiliations:** 1 Department of Biology, The Pennsylvania State University, University Park, PA, 16802, USA; 2 Penn State Neuroscience Institute, The Huck Institutes of the Life Sciences, University Park, PA, 16802, USA; 3 Center for Neural Engineering, The Pennsylvania State University, University Park, PA, 16802, USA; 4 Department of Biomedical Engineering, The Pennsylvania State University, University Park, PA, 16802, USA; 5 Department of Electrical and Electronics Engineering, Abdullah Gül University, Kayseri, Türkiye; 6 Department of Engineering Science and Mechanics, The Pennsylvania State University, University Park, PA, 16802, USA; 7 Department Neurosurgery, The Pennsylvania State University, University Park, PA, 16802, USA

**Keywords:** adolescent, alcohol, DID, prefrontal, aversion-resistant drinking, functional connectivity, fMRI

## Abstract

The prefrontal cortex (PFC) is one of the last brain regions to fully mature, making it particularly sensitive to stress and drug use early in life. Both human and rodent studies find long-lasting behavioral changes after adolescent alcohol exposure that implicate underlying disruptions in PFC development, including structural abnormalities and altered brain functional connectivity. Few rodent studies have been conducted to understand the network-level implications of these disruptions. We assessed how adolescent binge-like alcohol consumption in a drinking in the dark (DID) model affected adult aversion-resistant alcohol consumption, exploration, and brain-wide functional connectivity in mice. Approximately one month after the conclusion of DID, only female mice exposed to alcohol during adolescence exhibited aversion-resistant alcohol preference in adulthood. Adult females exhibited additional sex-specific changes in exploratory behavior in the elevated plus maze after adolescent alcohol consumption. Resting state neuroimaging revealed changes in prefrontal cortical connections with sensory motor, hippocampal, striatal, and other networks, providing insights into the putative systems underlying deficits caused by adolescent alcohol exposure. Critically, our data corroborate a growing body of literature in human and rodent studies demonstrating that adolescent alcohol use may increase risk for adult alcohol use more strongly in females. Finally, we identify neural correlates of this effect that include both known and novel networks and tie these back to human datasets, allowing biological and mechanistic targets to be further explored for future study and interventions.

## 1. INTRODUCTION

Adolescent alcohol use is associated with increased adult alcohol use and increased risk of developing an alcohol-related disorder^1–3^. Recent data indicate that this risk persists throughout the lifetime, and these positive associations between adolescent and later life alcohol use are stronger in females than in males^3^. These and other lasting behavioral consequences of adolescent alcohol exposure are thought to be driven by disturbances in the natural development of both morphological and functional characteristics of the brain. Normative adolescent brain development requires selective pruning of neuronal connections and cortical thinning^4^, but adolescent alcohol use has been shown to disrupt rates of cortical thinning^5^. Complementary lines of evidence show disruptions in functional connectivity between the default-mode network and an intrinsic functional network implicated in regulating emotional responses^6^ as well as within cortical and striatal networks^7^, suggesting a potential network mechanism for the increased vulnerability seen to adult alcohol use disorder (AUD). Vulnerability to development of AUD can also be exacerbated by other psychiatric conditions, such as anxiety^8^, which is heightened after alcohol use^9^, suggesting reciprocal feedback between substance use and mood disorders^10^.

Despite the known behavioral consequences of adolescent binge drinking and novel insights into brain connectivity changes made via functional imaging, further work must be done to bridge the gap between human epidemiological studies and the controlled assessment of the relationship between total alcohol intake and behavioral and mechanistic biological brain changes conducted with rodent studies. Here, we examined the long-term consequences of voluntary alcohol consumption during adolescence in a mouse model. Male and female C57BL/6J mice received intermittent access to alcohol in a Drinking in the Dark (DID) paradigm^11^ throughout adolescence and were tested for changes in aversion-resistant alcohol preference, exploratory behaviors, and whole-brain resting state functional connectivity^12^ in adulthood. We found binge drinking-induced changes in alcohol preference, behaviors related to exploratory and anxiety phenotypes, and functional connectivity within a prefrontal polymodal association network, all of which showed marked sex differences. This data suggests alterations in prefrontal cortical networks induced by voluntary alcohol consumption may underlie adulthood shifts in risk-taking and executive-decision making behaviors, consistent with what has been proposed in the human literature^13^. Our current work further bridges the gap between human epidemiological and behavioral studies and preclinical mechanistic biological studies via a shared non-invasive imaging dataset and allows for synthesis across human and animal studies.

## 2. MATERIALS AND METHODS

### 2.1 Animals

Male and female C57BL/6J (JAX #000664) mice were bred in-house for experiments beginning during adolescence (Bar Harbor, ME, USA; see Supplemental Materials for adult experiments). Mice were weaned on postnatal day (PND) 21 and single-housed in a room with a reversed light cycle (lights off at 7:00 AM) for at least one week prior to experiments. Mice had ad libitum access to water and food outside of DID testing (LabDiet 5053, Lab Diet, St. Louis, MO, USA). During DID (described below), water bottles were replaced with alcohol bottles 2-4 hr. All procedures were conducted in accordance with the NIH Guide for the Care and Use of Laboratory Animals and were approved by the Penn State Institutional Animal Care and Use Committee.

### 2.2 Drinking in the Dark (DID)

DID was performed as described previously^11,14–16^. Beginning on PND 29 (+/−1 day) for adolescent experiments, mice had intermittent access to 20% v/v ethanol (EtOH; Koptec, Decon Labs, King of Prussia, PA) in H2O (tap water). EtOH access began 3 hr into the dark cycle. For the first 3 days, alcohol access lasted 2 hr, and on the fourth day, alcohol access lasted 4 hr. After 3 days of abstinence, the DID cycle was repeated for a total of 4 cycles, with the last binge day falling on PND 53 (+/−1 day). Ages differed for additional adult behavioral experiments (see Supplemental Materials). Control subjects received access to water. Mice were weighed once weekly.

### 2.3 Preference for quinine-adulterated EtOH

EtOH preference was measured as published previously^16,17^. On PND 77-80, mice began acclimation to sipper tubes containing H2O (tap water). Sipper tubes were weighed and refilled every 48-72 hr to monitor intake. Approximately 30+ days after DID (PND 84-87) mice underwent a two-bottle choice paradigm (2BC) where they received access to one tube of H2O and one tube of increasing concentrations of EtOH in H2O (3% w/v EtOH for 2 days, 7% w/v EtOH for 5 days, and 10% w/v EtOH for two weeks). After this acclimation procedure, the 10% w/v EtOH in tap water was mixed with increasing concentrations of quinine hydrochloride (0.03, 0.1, 0.3, and 1 mM). Positions of H2O and EtOH tubes were alternated after every refill to avoid side preferences. Preference for EtOH solutions was defined as (EtOH consumed / total fluid consumed). Occasional missing data points due to bottle leakage were omitted in preference analyses and imputed in analyses of total amounts consumed. Mice were weighed once weekly.

### 2.4 Open field testing (OFT) and elevated plus maze (EPM)

Behavior in the open field test (OFT) and elevated plus maze (EPM) were assessed roughly 30 days (or 24 hr, see Supplemental Materials) after DID during the dark stage of the light cycle and under red light (8:00 AM-4:00 PM). Mice were transferred to a procedure room and acclimated for one hr prior to behavior testing. First, mice were placed in the corner of an open field box (50 × 50 cm; walls were 20 cm black plexiglass) and allowed to explore for 5 min^18–20^. After OFT testing, mice were returned to home cages in the procedure room and allowed to acclimate again for at least one hour before EPM testing. Then, mice were placed in the center of an elevated plus maze (30 × 5 cm arms; walls were 20 cm clear plexiglass on closed arms; maze was elevated 40 cm) and allowed to explore for 5 min^18^. EPM data were excluded after mice occasionally fell off of the maze (three times for primary experiment 30 days after adolescent DID).

### 2.5 Functional magnetic resonance imaging (fMRI)

#### Acclimation and data acquisition:

Mice were acclimated to the imaging environment for 4 days prior to testing. Briefly, mice were anesthetized with 3% isoflurane while their limbs were lightly bound using surgical tape. Mice were gently positioned in a cylindrical restrainer tube and secured with a bite bar. After mice awoke from anesthesia, they were monitored while acclimating for increasing amounts of time. Specifically, mice were restrained for 15, 30, 45, and 60 min on acclimation days 1-4, respectively, while a soundtrack of fMRI noises was played. Afterward, mice were anesthetized again before being removed from the restrainer and being unbound. On PND 83-88 (at least 30 days after DID), awake resting state imaging was then performed using a 7T MRI system and Bruker Console (Billerica, MA) using Paravision 7.0.0. Again, mice were briefly anesthetized with 3% isoflurane while being restrained. Once restrained, anesthesia was stopped and mice woke up before imaging. A gradient-echo echo-planar imaging (GE-EPI) sequence for T2*-weighted resting-state scans was used for data collection (repetition time (TR) = 1.5 s; echo time (TE) = 13.8 ms; field of view (FOV) = 1.6 × 1.6 cm; image matrix size 64 x 64; slice number = 16; slice thickness 0.75 mm; slice gap = 0 mm). Each scan lasted 10 min and collected 400 volumes, with three scans per mouse. Rapid imaging with refocused echoes (RARE) was used to acquire T1-weighted anatomical images (TR = 1.5 s; TE = 8 ms; FOV = 1.6 × 1.6 cm; image matrix size = 128 × 128; slice number = 16; slice thickness = 0.75 mm; repetition number = 6; slice gap = 0 mm).

#### Image preprocessing:

Framewise displacement (FD) was calculated to quantify motion^21^: FD = ∣ Δx ∣ + ∣ Δy ∣ + ∣ Δz ∣ + r * (∣ Δα ∣ + ∣ Δβ ∣ + ∣ Δγ ∣). r = 2 mm represents the length from the cortex to the center of the head. Δx, Δy, and Δz (translation distances) and Δα, Δβ, and Δγ (rotations) across x, y, and z axis were calculated using “imregtform” geometric transformation function in MATLAB version R2023b (The MathWorks Inc., Natick, MA, United States). Volumes exhibiting motion over half of the in-plane voxel size (0.125mm) and their temporally adjacent volumes were removed from analysis. Data were manually co-registered to a reference template for anatomical assessment and normalized to Allen Mouse Brain Atlas^22^ using a custom-developed MATLAB program^12^. Motion calculation and correction were performed using the Statistical Parametric Mapping (SPM12) package (http://www.fil.ion.ucl.ac.uk/spm/). We performed spatial smoothing using a Gaussian kernel (full-width-half-maximum = 0.375mm, 1.5x the x-y plane voxel size). We performed nuisance regression to eliminate signals originating from white matter (WM) and ventricle voxels, thereby reducing physiological noise (respiration, heart rate, etc.), along with regressing out the six motion parameters for each fMRI frame. Finally, a band-pass filter (0.01-0.1 Hz) was used. For consistency across data sets, 350 volumes from each scan (originally containing 400 volumes) were used.

#### Image postprocessing:

A total of 62 regions of interest (ROIs) were defined based on definitions from the Allen Mouse Brain Atlas^22^. ROIs were assigned to 10 brain systems: hippocampal region, retrohippocampal region, striatum, sensory-motor cortex, polymodal association cortex, olfactory cortex, pallidum, thalamus, hypothalamus, and midbrain. Resting state functional connectivity between brain regions was analyzed using an ROI-based approach specifically testing Pearson correlations between the average activity time course between each ROI pair. Functional connectivity matrices were calculated using Fisher’s z-transformed Pearson’s correlation coefficients within each mouse. Seed-based functional connectivity analysis was also used to directly determine regions with strong functional connectivity with the prefrontal cortex.

### 2.6 Statistics

#### Behavioral analysis:

Statistical analysis was performed in GraphPad Prism (La Jolla, CA, USA) and MATLAB version 9.10 (R2021a; The MathWorks Inc., Natick, MA, United States) and figures were made in GraphPad Prism. A significance threshold of α = 0.05 was used for all analyses. DID drinking values that exceeded 2 g within 2 hr were categorized as leaks and removed from drinking analyses. Outliers for adult outcomes were identified in the ROUT outlier test (Q=1%), and mice had to be outliers across more than one measurement within the same test to be excluded. No mice met these criteria in Figs 2–3. Two- and three-way ANOVA were used to analyze outcome variables across all mice, using a mixed model for repeated measures. Sex and alcohol interactions were followed up with t tests between alcohol and control groups within each sex. Additional analyses explored the relationship between total alcohol consumption and adult outcomes using generalized linear mixed-effects (GLME) modeling in alcohol-exposed mice. Accounting for litter and behavioral cohort as random effects, the model was defined as: [outcome variable] ~ 1 + Sex*EtOH + (1|Litter) + (1|Cohort). For these analyses, sex and alcohol interactions were followed up with additional GLME models within each sex: [outcome variable] ~ 1 + EtOH + (1|Litter) + (1|Cohort).

#### fMRI analysis:

Statistical analysis was performed and figures were made in MATLAB version R2023b (The MathWorks Inc., Natick, MA, United States). Runs with over 10% of volumes discarded due to motion artifacts were excluded from final analysis. The effects of adolescent alcohol consumption, sex, and their interaction on functional connectivity was assessed using a linear mixed model, with subject variability treated as a random effect. The effects with p<0.05 are regarded as significant.

## 3. RESULTS

### 3.1 Adolescent binge drinking led to minor differences in sex-specific physiological growth

**Figure 1:**
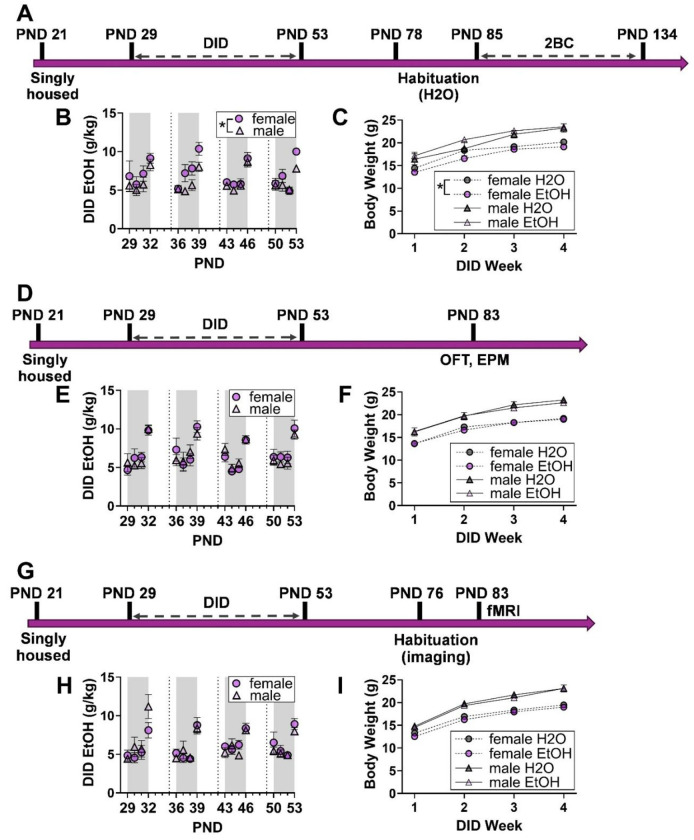
Experimental timelines, adolescent alcohol consumption, and body weights across cohorts. Three separate experiments tested the influence of adolescent drinking in the dark (DID) on adult aversion-resistant alcohol preference via two-bottle choice (2BC; **A-C**), exploratory behavior in the open field test (OFT) and elevated plus maze (EPM; (**D-F**), and whole brain resting state functional connectivity (**G-I**). PND is approximate (see methods). Data are shown as mean +/− SEM. *p < 0.05. n = 9-11 (**A-C**), 8-10 (**D-F**), or 11 (**G-I**) per sex per treatment.

Adolescent male and female mice received intermittent access to alcohol in a DID paradigm across all experiments. Daily alcohol consumption was analyzed via two-way (sex, day) mixed model ANOVA and body weights were analyzed via three-way (treatment, sex, week) mixed model ANOVA. In the first experimental cohort (Fig 1A), adolescent females consumed more alcohol than adolescent males (F_1,19_ = 10.2, *p* = 0.0048; Fig 1B). An interaction between treatment and sex was detected for body weight (F_1,33_ = 4.1, *p* = 0.0498; Fig 1C), and follow-up two-way ANOVA (treatment, week) within sexes revealed that alcohol-treated females weighed less than female water controls (F_1,18_ = 4.5, *p* = 0.0476) and alcohol exposure interacted with week (F_3,54_ = 3.2, *p* = 0.0309). No treatment effect was seen for male body weights. No differences were seen in alcohol consumption and body weight in the second (Fig 1D–F) and third (Fig 1 G–I) experimental cohorts. Additional sex and week effects on body weights are further described in Supplemental Materials.

### 3.2 Adult preference for alcohol and quinine-adulterated alcohol after adolescent drinking

Beginning approximately 30 days after adolescent drinking, alcohol preference and aversion-resistant alcohol consumption was tested. Adult mice received continuous access to a bottle of alcohol containing increasing concentrations of the bitter tastant quinine alongside a bottle of water (timeline in Fig 1A). Preference for alcohol, fluid consumption, and body weight were monitored. Because adolescent females consumed more alcohol than adolescent males in this cohort, and because there were significant sex-dependent interactions between alcohol and physiological growth, adult males and females were analyzed separately for categorical (EtOH vs H2O groups) analyses. Preference, fluid consumption, and body weight were analyzed via two-way (treatment, week) mixed model ANOVA.

**Figure 2:**
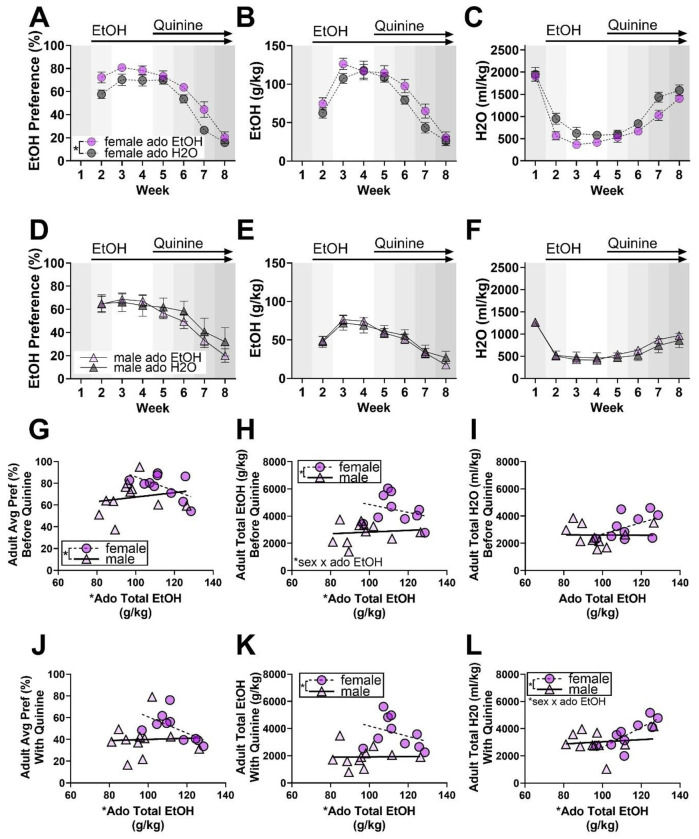
Adolescent alcohol consumption increased adult preference for alcohol and quinine-adulterated alcohol in females and not in males. **(A)** EtOH preference in females. **(B)** EtOH g/kg consumed by females. **(C)** Water consumed in ml/kg by females. **(D)** EtOH preference in males. **(E)** EtOH g/kg consumed by males. **(F)** Water consumed in ml/kg by males. **(G)** Average EtOH preference compared to adolescent EtOH consumption in females. **(H)** Total EtOH consumption during 2BC in g/kg compared to adolescent EtOH consumption by females. **(I)** Total water consumption during 2BC in ml/kg compared to adolescent EtOH consumption in females. **(J)** Average EtOH preference compared to adolescent EtOH consumption in males. **(K)** Total adult EtOH consumption during 2BC in g/kg compared to adolescent EtOH consumption in males. **(L)** Total water consumption during 2BC in ml/kg compared to adolescent EtOH consumption in males. Ado = Adolescent. Week 1 represents consumption of H2O only, Week 2 includes 3-7% EtOH, Week 3-4 represents 10% EtOH, and Weeks 5-8 represent 10% EtOH with increasing concentrations of quinine (0.03, 0.1, 0.3, and 1mM). Plots labeled as “Before Quinine” represent data from Weeks 1-4, and plots labeled as “With Quinine” represent data from Weeks 5-8. In scatter plots, a simple linear regression was used to generate lines of best fit and aid interpretation but were independent from statistical analysis. Data are shown as mean +/− SEM. * *p* <0.05. n=9-11/sex/treatment.

Adult females that consumed alcohol during adolescence exhibited stronger preference for alcohol and quinine-adulterated alcohol than females that only had water during adolescence (F_1,18_ = 4.7, *p* = 0.0441; Fig 2A). Female alcohol preference differences were likely driven by a reciprocal increase in alcohol and decrease in water consumption, as adult female alcohol, water, and total fluid consumption by themselves were not significantly altered by adolescent alcohol exposure (Fig 2B–C, Supplemental Materials). Adult female body weight was also not impacted by adolescent alcohol exposure (Supplemental Materials). In adult males, adolescent alcohol consumption did not significantly influence adult alcohol preference, alcohol consumption, water consumption, total fluid consumption, or body weight (Fig D-F, Supplemental Materials). Across females and males, all measures varied by week (F Pref: F_4,70_ = 109.1, p < 0.0001; F EtOH: F_5,81_ = 99.42, p < 0.0001; F H2O: F_4,75_ = 124.5, p < 0.0001; F Fluid: F_4,72_ = 4.5, p = 0.0028; F Weight: F_4,74_ =, p < 0.0001; M Pref: F_3,58_ = 27.0, p < 0.0001; M EtOH: F_3,58_ = 43.7, p < 0.0001; M H2O: F_4,68_ = 39.9, p < 0.0001; M Fluid: F_3,60_ = 8.4, p < 0.0001; M Weight: F_2,35_ = 41.9, p < 0.0001). Overall, adolescent alcohol consumption increased adult preference for alcohol and quinine-adulterated alcohol in females and not in males.

To directly compare sexes while accounting for differences in adolescent alcohol consumption, we used a GLME model. Before quinine was added to alcohol (weeks 2-4), average alcohol preference was inversely associated with total alcohol consumption (ado EtOH: −0.0092989 ± 0.0040242, p = 0.0337) and was lower in males than in females (quinine male: −1.22 ± 0.51557, p = 0.0301; Fig 2G). Similar effects of total adolescent alcohol and sex were seen on total adult alcohol consumption. Adolescent alcohol consumption was associated with decreased adult alcohol consumption (ado EtOH: −77.03 ± 19.392, p = 0.0010) and males consumed less alcohol without quinine than females (male: −7300.1 ± 2334.3, p = 0.0061; Fig 2H). Total adult alcohol consumption without quinine was also influenced by an interaction of sex and adolescent alcohol (male x ado EtOH: 46.756 ± 21.202, p = 0.0415). Follow-up analyses within sexes confirmed an effect of adolescent alcohol in males (ado EtOH: −20.486 ± 8.4987, p = 0.0392) but not in females. Adult total water consumption prior to quinine testing was not altered by sex or adolescent alcohol consumption (Fig 2I). In short, adolescent alcohol consumption was inversely associated with adult alcohol consumption and preference prior to quinine testing, and an interaction of adolescent alcohol with sex was noted for total quinine-free alcohol consumption.

Similar effects of total adolescent alcohol consumption and sex were seen on alcohol preference, alcohol consumption, and water consumption after quinine was added to the two-bottle choice test (weeks 5-8). Total adolescent alcohol consumption had an inverse association with average alcohol preference with quinine (ado EtOH: −0.010501 ± 0.0043085, p = 0.0261; Fig 2J), but sex effects on alcohol preference with quinine were not detected. Total adolescent alcohol consumption was associated with decreased adult alcohol consumption (ado EtOH: −85.924 ± 22.866, p = 0.0016) and adult males consumed less alcohol than adult females with quinine (male: −7122.3 ± 2759.8, p = 0.0194; Fig 2K). Further, during quinine testing, adolescent alcohol consumption was positively associated with adult water consumption (ado EtOH: 73.618 ± 23.207, p = 0.0056), adult males consumed more water than adult females (male: 6981.3 ± 3163.4, p = 0.0414), and sex and adolescent alcohol consumption interacted to influence adult water consumption (male x ado EtOH: −65.655 ± 29.139, p = 0.0377; Fig 2L). Follow-up analyses within sexes found a significant positive effect of adolescent alcohol consumption on adult water consumption during quinine testing in females (ado EtOH: 73.618 ± 19.258, p = 0.0051) but not in males. Additionally, total fluid consumption and average body weight across all adult testing weeks were not impacted by sex or total adolescent alcohol consumption (Supplemental Materials). To summarize, total adolescent alcohol consumption had an inverse association with average adult preference and alcohol consumption during the quinine test (weeks 5-8). Overall changes in fluid consumption were not observed, suggesting a specific effect of adolescent alcohol consumption on adult alcohol preference and not generalized consummatory behaviors. These data support a nuanced relationship between adolescent alcohol exposure and adult alcohol use.

### 3.3 Adult exploration in the OFT and EPM after adolescent drinking

In a separate cohort of mice, approximately 30 days after adolescent drinking, we tested adult exploratory behaviors in the OFT and EPM (Fig 1D). Because adolescent mice of this cohort consumed comparable amounts of alcohol, males and females were includes in the same categorical (EtOH vs H2O groups) two-way (treatment, sex) ANOVA. Additional GLM analyses tested the relationship between total adolescent alcohol consumption and adult behavior among alcohol-exposed subjects.

**Figure 3:**
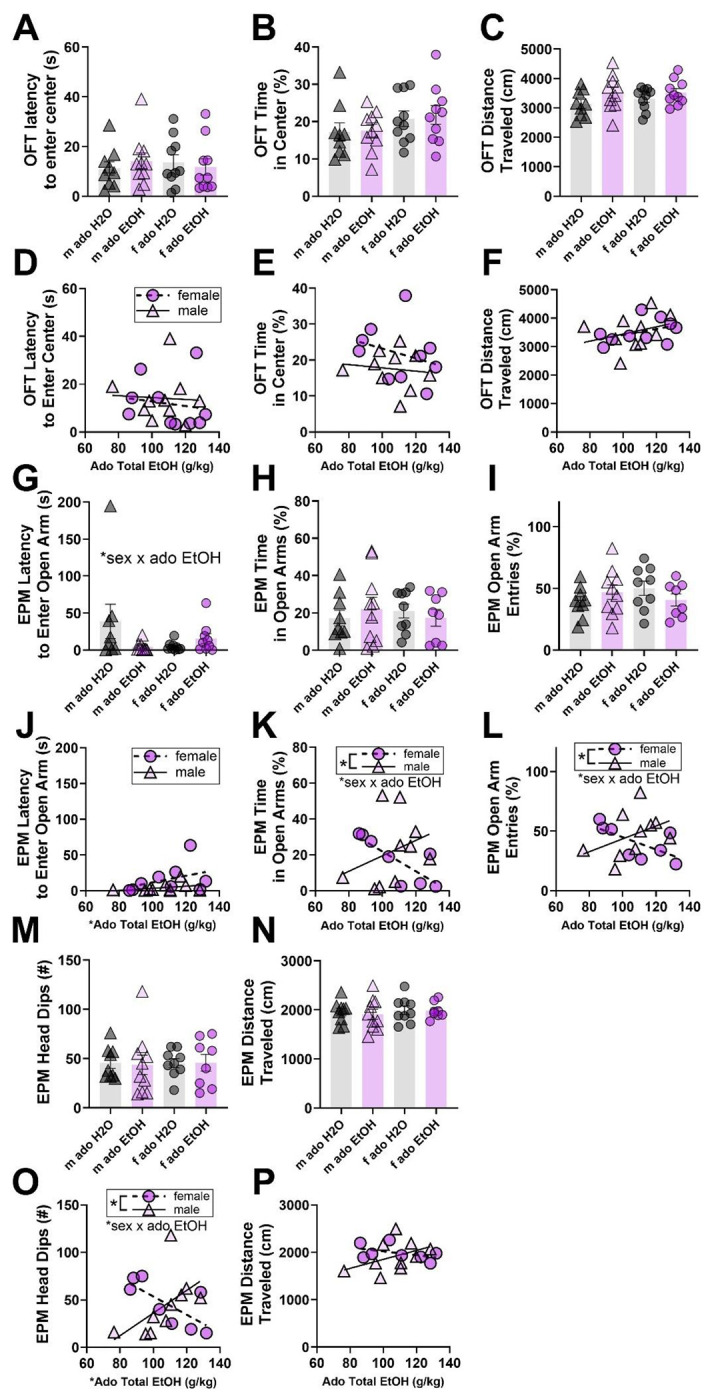
Adolescent alcohol consumption had sex-specific effects on adult exploratory behaviors. **A)** OFT latency to enter center of arena. **(B)** OFT percent time spent in center of arena. **(C)** OFT total distance traveled. **(D)** OFT latency to enter center compared to adolescent EtOH consumption. **(E)** OFT percent time in center compared to adolescent EtOH consumption. **(F)** OFT total distance traveled compared to adolescent EtOH consumption. **G)** EPM latency to enter an open arm. **(H)** EPM percent time spent in open arms. **(I)** EPM percent open arm entries. **(J)** EPM latency to enter an open arm compared to adolescent EtOH consumption. **(K)** EPM percent time spent in open arms compared to adolescent EtOH consumption. **(L)** EPM percent open arm entries compared to adolescent EtOH consumption. **M)** EPM number of head dips. **(N)** EPM total distance traveled. **(O)** EPM number of head dips compared to adolescent EtOH consumption. **(P)** EPM total distance traveled compared to adolescent EtOH consumption. M = male; f = female; ado = adolescent. In scatter plots, a simple linear regression was used to generate lines of best fit and aid interpretation but were independent from statistical analysis. Data are shown as mean +/− SEM. *p<0.05. n=8-10/sex/treatment.

First, mice were placed in an open field chamber and video recorded for 5 min. No effects of sex or adolescent alcohol treatment group were detected for latency to enter the center (Fig 3A), time spent in the center (Fig 3B), or overall distance traveled (Fig 3C). Similarly, total adolescent alcohol consumed was not predictive of adult latency to enter the center (Fig 3D), time spent in the center (Fig 3E), or distance traveled in the open field in an analysis of only alcohol-exposed mice (Fig 3F).

Next, mice underwent testing in the elevated plus maze and behavior was monitored over 5 min. Adolescent alcohol consumption and sex significantly interacted to alter latency to enter an open arm of the maze (F_1,32_ = 4.4, p = 0.0446; Fig 3G), though follow-up t tests within sexes did not reveal differences across treatment groups. Time spent in open arms (Fig 3H) and number of open arm entries (Fig 3I) were not different across adolescent alcohol or sex groups. Additional analyses tested the associated between total adolescent alcohol consumption and adult behaviors in only alcohol-exposed mice, and no effects were seen for latency to enter EPM (Fig 3J), but this revealed an interaction of adolescent alcohol consumption and sex on time spent in the open arm (male x ado EtOH: 0.9827 ± 0.4244, p = 0.0363; Fig 3K) and open arm entries (male x ado EtOH: 1.0538 ± 0.4220, p = 0.0256; Fig 3L). Follow-up analyses within sexes revealed significant reductions in exploratory behaviors due to total alcohol exposure within females, and not males, as indicated by reduced time spent in the open arm (ado EtOH: −0.6544 ± 0.0290, p < 0.0001) and open arm entries (ado EtOH: −0.5535 ± 0.1947, p = 0.0295). Further, total adolescent alcohol consumption alone was significantly associated with latency to enter the open arm (ado EtOH: 0.0463 ± 0.01146, p = 0.0011). Sex was significantly associated with time spent in the open arms (male: −100.69 ± 45.956, p = 0.0459) and entries into open arms (male: −106.73 ± 45.694, p = 0.0349). Further, number of head dips (Fig 3M) and total distance traveled (Fig 3N) were not significantly affected by adolescent alcohol group or sex. In follow-up analyses testing effects of total alcohol consumed, a significant interaction of adolescent alcohol consumption and sex was detected for head dips (male x ado EtOH: 2.1466 ± 0.6536, p = 0.0054) in addition to a significant influence of total adolescent alcohol exposure (ado EtOH: −0.9572 ± 0.4410, p = 0.0477) and sex (male: −232.09 ± 70.78, p = 0.0055; Fig 3O). Follow-up analyses within sexes showed significant reductions in head dips due to total alcohol exposure within females, and not males (ado EtOH: −1.0553 ± 0.2346, p = 0.0041). No effects of sex or total alcohol consumed were seen for distance traveled in the EPM (Fig 3P).

In summary, EPM testing revealed sex-specific exploratory deficits due to adolescent alcohol consumption. Across multiple measures, total adolescent alcohol consumption decreased exploratory behavior in females and not in males. These findings contrast those seen 24 hours after adolescent DID and 30 days after adult DID (Supplemental Materials), suggesting that behavioral effects of DID are specific to adolescent exposures and duration of alcohol abstinence post-DID.

### 3.4 Adolescent binge drinking led to sex-specific alterations in adult whole-brain functional connectivity and pathway-specific connectivity

**Figure 4.**
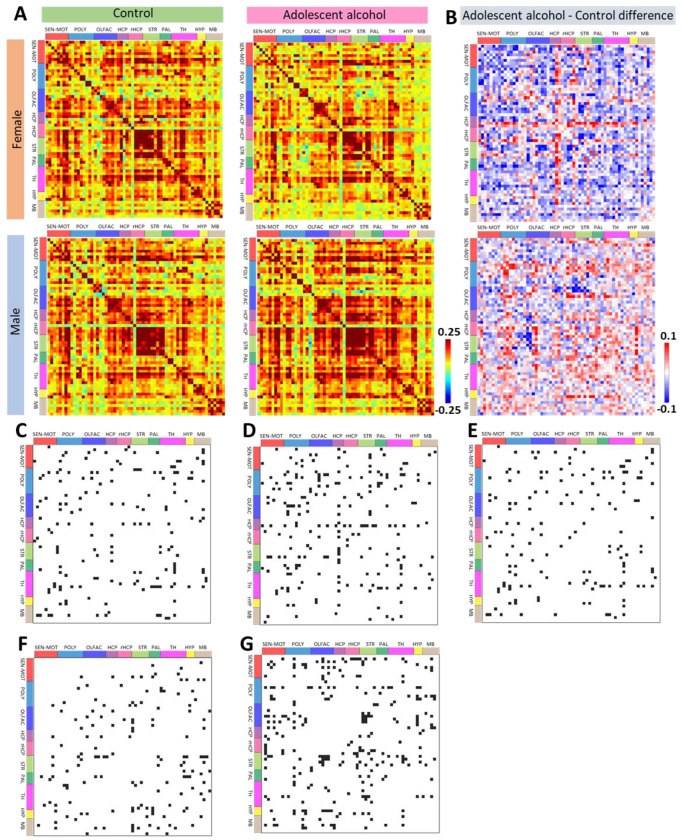
Whole brain functional connectivity differs across sex and due to adolescent alcohol consumption. **(A)** Average resting state functional connectivity (RSFC) matrices for sex and alcohol groups. The color bar (± 0.25 range) represents the z-score values for the corresponding RSFC. **(B)** Difference of RSFC between alcohol and control groups for each sex. The red-blue color bar (± 0.1 range) shows the z-score differences for the increased or decreased average RSFC in adolescent alcohol-exposed mice in comparison to the controls. Significant effects of adolescent alcohol group **(C)**, sex **(D)**, and the interaction between adolescent alcohol group and sex **(E)** were found across pairwise resting state functional connectivity (RSFC). **(F)** Among alcohol-exposed mice, significant effects of total alcohol consumption were found on each pairwise RSFC after accounting for sex. **(G)** Significant effects of sex were also found on each pairwise RSFC after accounting for total alcohol consumption (Linear mixed model effect, p<0.05, FDR uncorrected).

A final cohort of mice underwent resting state functional magnetic resonance imaging (rs-fMRI) roughly 30 days after adolescent drinking (Fig 1C). The mouse brain was partitioned to 62 bilateral regions of interest (ROIs) anatomically defined in the Allen Mouse Brain Atlas^22^ (see Supplemental Materials for ROI descriptions). We saw effects of alcohol consumption, sex, and interactions across a variety of measurements. Females showed a strong brain-wide decrease in overall global functional connectivity post-binge drinking (Fig 4A–B). In contrast, males showed a brain-wide increase in overall global functional connectivity post-binge drinking (Fig 4A–B). We further assessed this data using linear mixed models to account for repeated scans and individual alcohol consumption levels. We saw significant effects of treatment group, total alcohol consumed, sex, and interactions of alcohol and sex across a multitude of brain regions (Fig 4C–G, Supplemental Materials).

**Figure 5.**
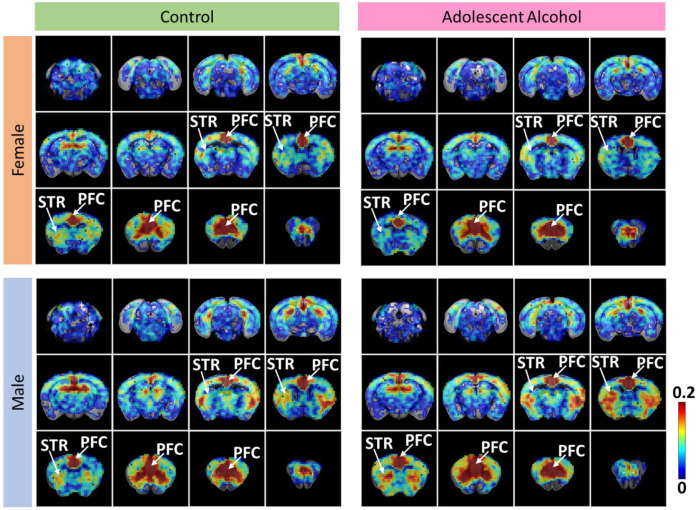
PFC seed-based functional connectivity shows differences in connectivity with key addiction regions, such as the striatum. Average seed-based functional connectivity maps of PFC for sex- and treatment-specific groups. Anatomical classification of PFC is available in Supp Fig 5. The color bar (± 0.2 range) represents the average correlation values for the corresponding RSFC between the PFC and all other voxels. PFC and the groups of brain voxels detected as striatum (STR) are indicated with white arrows.

Based on the known functional connectivity deficits seen following substance use and our a-priori hypotheses, we further explored this data with the PFC as a seed region (Fig 5, Supplemental Materials). Here, we saw that female mice showed a decrease in functional connectivity (Fig 5), and male mice showed an increase in functional connectivity (Fig 5), between the PFC and both dorsal and ventral striatum.

Together, functional imaging data demonstrates a robust sex-dependent and dose-dependent alteration in whole brain connectivity and PFC connectivity, consistent with sex differences in behavioral profiles.

## 4. DISCUSSION

This series of studies tested the relationship between voluntary adolescent alcohol consumption and adult behaviors and whole-brain functional connectivity changes in a mouse model of binge drinking. Adolescent C57BL/6J mice reliably consumed binge levels of alcohol, consistent with our previously published work^15,16^. Beginning approximately one month after adolescent DID, we found that adult females with a history of alcohol consumption had a greater preference for alcohol and quinine-adulterated alcohol than water-exposed controls. Dose effects of adolescent alcohol exposure were complex, as total alcohol consumed in adolescence had a significant negative association with adult alcohol preference. Additional tests examined adult exploratory behaviors in the OFT and EPM after adolescent alcohol exposure, and these analyses revealed complementary sex-specific changes in exploration that were also sensitive to adolescent alcohol dose. Finally, fMRI experiments revealed alterations in prefrontal cortical-driven polymodal association network connectivity with brain regions known to be involved in AUD in adult humans, such as the striatum, as well as provided a rich dataset of novel networks influenced by adolescent alcohol consumption and biological sex. Cumulatively, these data provide evidence of long-term disruptions in addiction-related behaviors and brain function attributable to adolescent alcohol exposure, particularly in females, and provides a bridge between biologically-intensive mechanistic animal studies and human work.

This study examined the relationship between adolescent alcohol consumption and adult aversion-resistant alcohol preference. This was motivated by our prior work demonstrating that despite profound changes in prefrontal cortical function following adolescent alcohol consumption^15^, adolescent consumption does not robustly increase adult alcohol consumption across multiple rodent models^16^. Here, we extend these foundational studies to find persistently elevated adult alcohol preference despite a quinine adulteration in female, and not male, mice following adolescent DID. This suggests that adding a challenge and/or changing the components of the adult alcohol preference test reveals an addiction-like behavioral pattern in females that alcohol vs water two-bottle choice testing could not capture by itself. This is consistent with the framework that adolescent alcohol exposure impairs adult behavioral flexibility, or the ability to modify behavior in response to changes in an environment^23,24^. Our prior data suggested that adolescent alcohol exposure leads to a temporary elevated adult alcohol preference that subsides. Here we also find this transient increase in adult alcohol preference, but effects of adolescent alcohol consumption are revealed again in the presence of an aversive stimulus during adult drinking. This has clear implications for human alcohol use, as a key component of human AUD is the continued use of alcohol despite negative consequences^25^. Our data are further consistent with recent human data that demonstrated the positive relationship between adolescent binge drinking and later life alcohol use is dramatically stronger in females than in males^3^.

When total alcohol consumed during adolescence was compared to adult alcohol consumption and preference, we observed that lower adolescent alcohol consumption corresponded to increased adult alcohol consumption. While surprising, this is consistent with our prior data^16^. One possible interpretation is that our outcome variable, adult alcohol consumption, has an inverted U-shaped response curve, whereby adolescent mice may consume such high levels of alcohol in a DID paradigm that dose effects appear to have a negative slope. This may explain some of the inconsistent behavioral effects of different adolescent alcohol exposure paradigms across the literature^26^.

Additionally, we report that adolescent alcohol exposure had sex-specific effects on adult exploratory behavior in the EPM. Specifically, adolescent alcohol exposure increased exploration in males while decreasing exploration in females, and this scaled predictably with volume of adolescent alcohol consumed. Female-specific decreases in exploration coupled with female-specific susceptibility to aversion-resistant drinking accumulate to indicate that females with a history of adolescent alcohol use may be vulnerable to substance use and changes in general executive functioning through multiple mechanisms. This sheds light on the growing rate of AUD diagnoses in women^27^ and calls for more sex-specific research on AUD vulnerability. However, it is worth noting that prior studies using different adolescent alcohol protocols have reported mixed effects on adult EPM behaviors, with some reporting increased^28^, decreased^23^, or no changes^29–31^ in adult exploratory behaviors. This suggests that changes in exploratory behaviors due to adolescent alcohol exposure could be sensitive to specific experimenter-induced and designed experimental conditions, a phenomenon that could be particularly pronounced for EPM^32^.

Analyses of adolescent alcohol consumption showed reliable binge-like alcohol consumption, as our laboratory has published previously^15,16^. In one cohort, we observed significantly elevated alcohol consumption in females. This is not necessarily surprising because the literature provides robust support for adult females consuming more alcohol than adult males^11,33^. However, it is likely that pubertal onset and the beginning of adult hormonal fluctuations contribute to the development of elevated alcohol consumption in females, and the timing of this can vary within individuals^34^. This, combined with the small effect size of the observed sex difference in adolescent alcohol consumption, suggests true sex differences in alcohol consumption may not emerge until post puberty, and varying differences in consumption by biological sex seen across cohorts represent this fluctuating onset.

Whole-brain functional connectivity was assessed to identify changes in neural circuits underlying changes in alcohol preference and exploration. While females show decreased global brain-wide functional connectivity and males showed increased brain-wide functional connectivity following adolescent binge drinking, we primarily focused our attention and analysis on the PFC and the polymodal association network due to the robust preclinical and human literature on alcohol’s effects on the PFC. Broadly, we found an increase in functional connectivity between the PFC and striatum in males, and a decrease of this connectivity in females, following binge alcohol consumption. The anterior cingulate cortex connectivity to the striatum (specifically the lateral septal complex, caudoputamen, nucleus accumbens, and olfactory tubercle) all showed significant main effects from alcohol exposure. Importantly, human imaging studies on adolescent binge drinkers has also identified the significance of striatal and cortical projections^7,35^. Work by Jones et al.^7^ uses a repeated measures design to show impaired developmental maturation of the PFC, including the anterior cingulate cortex, in previously alcohol-naive subjects who participated in regular binge drinking, suggesting that underdevelopment and hypoactivity of the PFC during adolescence is a critical component of the risky decision making seen with drug use. Our previous *ex vivo* electrophysiology work supports this framework^15^, which we expand with whole brain imaging here.

Prior animal work has evaluated the links between adolescent alcohol use, behavioral flexibility, and brain functional connectivity^13^. A preclinical study by Gómez and colleagues^24^ evaluated the impact of an intermittent intragastric adolescent alcohol exposure paradigm on adult behavioral flexibility in an attentional set shifting task and brain functional connectivity (under sedation) within the same rats. In this experiment, they found reduced functional connectivity between multiple regions of interest (ROIs), including the prelimbic cortex and the nucleus accumbens, and severity of functional connectivity deficits were associated with reversal learning deficits within subjects in a mediation analysis. Additionally, females had more pronounced reversal deficits than males after adolescent alcohol exposure. Thus, there is a clear link between adolescent alcohol exposure’s effects on functional connectivity and behavioral flexibility, and the behavioral deficits are consistently more severe in females. These findings are consistent with ours, suggesting that female-specific impairments in behavioral flexibility might also manifest or be related to increased aversion-resistant drinking in adulthood. While our current study used different subjects for behavioral and fMRI experiments, we used a voluntary alcohol consumption model that allowed for detection of dose effects of adolescent alcohol exposure on functional connectivity, and the experimental design did not require sedation during imaging. Our preclinical rodent model data therefore support the overall human imaging and cognitive processing framework of how adolescent brain function and structure matures over development, and the threat substances of abuse pose for these processes^13^.

To summarize, we found that adolescent binge-like alcohol consumption led to increased aversion-resistant drinking in females, sex-specific changes in exploratory behaviors, and changes in whole brain functional connectivity. These data suggest that females may be more vulnerable to lasting addiction-related behavioral consequences of adolescent alcohol exposure, and these may be driven by functional connectivity changes in prefrontal-striatal circuits. By using a voluntary adolescent alcohol consumption model, we were able to demonstrate nuanced dose effects, supporting that adolescent alcohol exposure can lead to a range of adult phenotypes. Further, this study provides biological targets for future studies evaluating the growing risk of alcohol use and misuse in women.

## Supplementary Material

1
