# Supplementary material for: Adolescent alcohol consumption alters sex-specific behaviors associated with prefrontal functional connectivity in mice
Source: bioRxiv. 2025 Jun 8:2025.06.05.658112. Preprint. [Version 1] doi: 10.1101/2025.06.05.658112 (PMC12258943; doi:10.1101/2025.06.05.658112)
Supplement: 1 [file NIHPP2025.06.05.658112v1-supplement-1.pdf]

bioRxiv preprint doi: https://doi.org/10.1101/2025.06.05.658112; this version posted June 8, 2025. The copyright holder for this preprint (which was not certified by peer review) is the author/funder, who has granted bioRxiv a license to display the preprint in perpetuity. It is made available under aCC-BY 4.0 International license.

Adolescent alcohol consumption alters sex-specific behaviors associated with prefrontal functional connectivity in mice

Running title: adolescent alcohol behavior and imaging

Seemiller et al.

Supplementary Materials

Supplementary Methods:

OFT and EPM 24 hr after adolescent DID

As described in the main text, male and female C57BL/6J mice were bred in-house, weaned and moved into single housing on a reverse light cycle on PND 21, and underwent DID PND 29-53 (+/- 1 day). One day after the conclusion of DID (PND 53 +/- 1), mice began OFT and EPM testing. One subject jumped out of the OFT box during testing, so OFT time in center and distance traveled were not collected for that mouse. Statistical analysis and outlier testing was done as described in the main text. No outliers were detected for OFT or EPM data sets.

OFT and EPM 30 days after adult DID

For adult experiments, male and female C57BL/6J mice were ordered from Jackson Laboratory (Bar Harbor, ME, USA). At least one week prior to DID, subjects were moved and singly housed in a reversed light cycle room. DID was conducted as described in the main text, except that it occurred during adulthood (PND 62-86). No mice fell off the EPM. OFT and EPM testing began on PND 118. Statistical analysis and outlier testing was performed as described in the main text. One outlier subject was removed from EPM analysis.

Supplementary Results:

3.1 Adolescent binge drinking led to minor differences in sex-specific physiological growth

During adolescent DID, alcohol consumption across all cohorts was affected by day (Cohort 1: $F_{6,106} = 8.1$, $p < 0.0001$; Cohort 2: $F_{7,129} = 11.3$, $p < 0.0001$; Cohort 3: $F_{15,150} = 10.8$, $p < 0.0001$). Females weighed less than males (Cohort 1: $F_{1,33} = 37.4$, $p < 0.0001$; Cohort 2: $F_{1,36} = 42.2$, $p < 0.0001$; Cohort 3: $F_{1,40} = 71.6$, $p < 0.0001$) and body weights changed across weeks (Cohort 1: $F_{2,58} = 214.5$, $p < 0.0001$; Cohort 2: $F_{2,76} = 289.8$, $p < 0.0001$; Cohort 3: $F_{2,77} = 462.8$, $p < 0.0001$). Body weights were also significantly impacted by week in the sex-specific analysis of cohort 1 (female: $F_{2,43} = 300.1$, $p < 0.0001$; male: $F_{2,24} = 62.8$, $p < 0.0001$). Further, body weights were influenced by an interaction of week and sex (Cohort 1: $F_{3,99} = 3.1$, $p = 0.0308$; Cohort 2: $F_{3,108} = 3.8$, $p = 0.0129$; Cohort 3: $F_{3,120} = 8.9$, $p < 0.0001$). *Data are represented in **Fig 1** of the main text.*

bioRxiv preprint doi: https://doi.org/10.1101/2025.06.05.658112; this version posted June 8, 2025. The copyright holder for this preprint (which was not certified by peer review) is the author/funder, who has granted bioRxiv a license to display the preprint in perpetuity. It is made available under aCC-BY 4.0 International license.

3.2 Adult preference for alcohol and quinine-adulterated alcohol after adolescent drinking

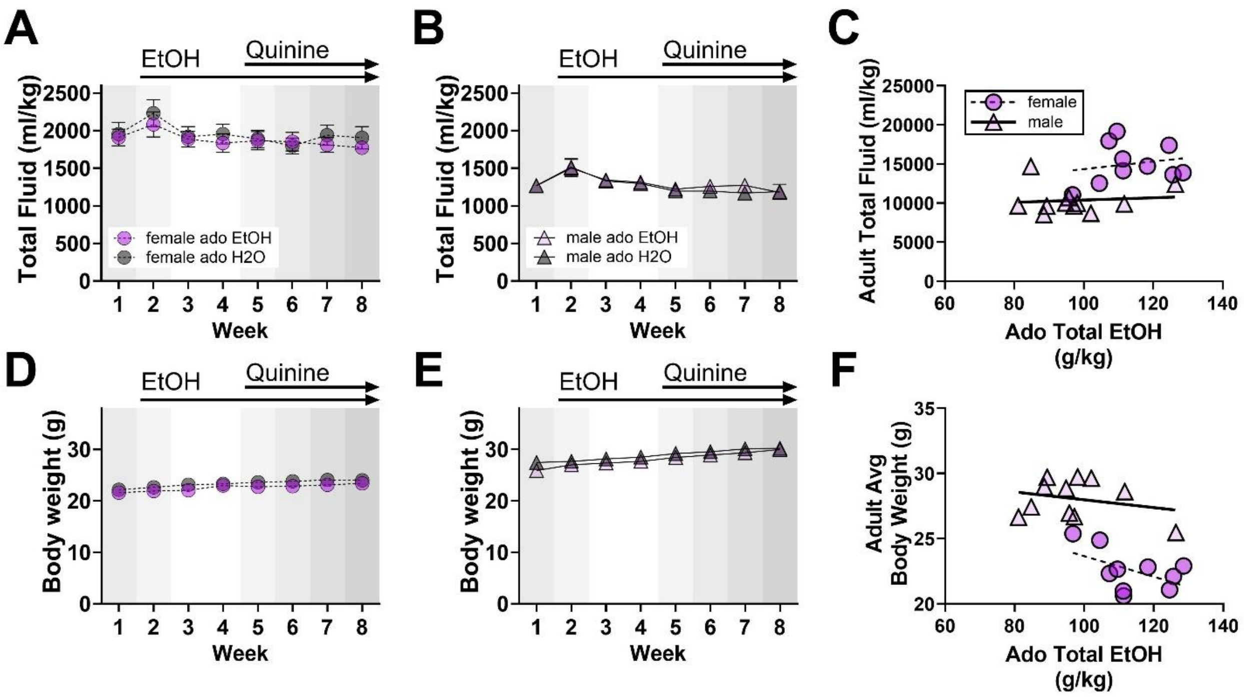

Supp Fig 1. Adolescent alcohol consumption did not affect total fluid consumption (**A-C**) or body weight (**D-F**) during two-bottle choice assessment of alcohol preference. Ado = Adolescent. Week 1 represents consumption of H2O only, Week 2 includes 3-7% EtOH, Week 3-4 represents 10% EtOH, and Weeks 5-8 represent 10% EtOH with increasing concentrations of quinine (0.03, 0.1, 0.3, and 1mM). Plots labeled as “Before Quinine” represent data from Weeks 1-4, and plots labeled as “With Quinine” represent data from Weeks 5-8. In scatter plots, a simple linear regression was used to generate lines of best fit and aid interpretation but were independent from statistical analysis. Data are shown as mean +/- SEM. * p <0.05. n=9-11/sex/treatment.

bioRxiv preprint doi: https://doi.org/10.1101/2025.06.05.658112; this version posted June 8, 2025. The copyright holder for this preprint (which was not certified by peer review) is the author/funder, who has granted bioRxiv a license to display the preprint in perpetuity. It is made available under aCC-BY 4.0 International license.

3.3 Exploration in the OFT and EPM after drinking

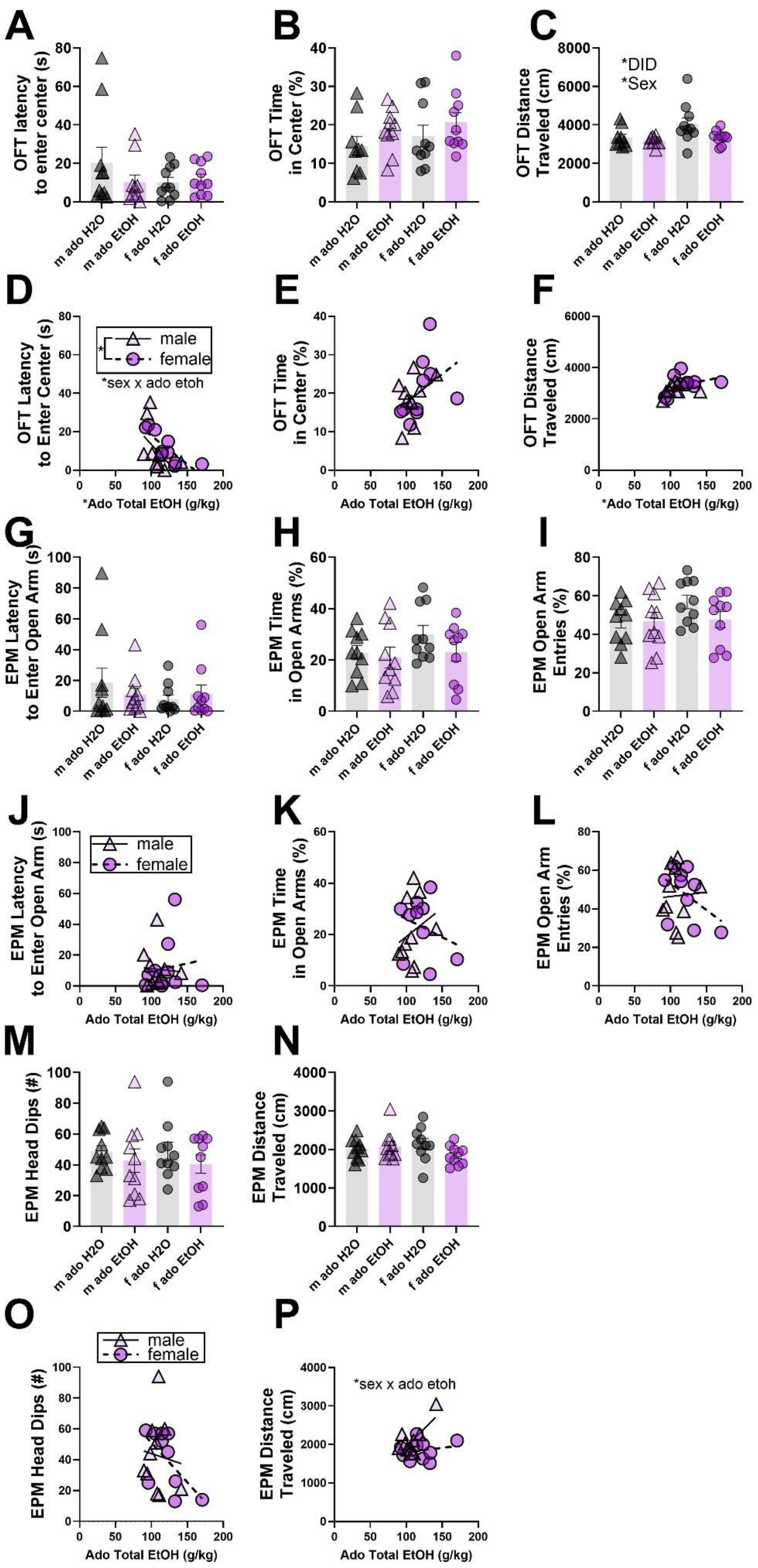

bioRxiv preprint doi: https://doi.org/10.1101/2025.06.05.658112; this version posted June 8, 2025. The copyright holder for this preprint (which was not certified by peer review) is the author/funder, who has granted bioRxiv a license to display the preprint in perpetuity. It is made available under aCC-BY 4.0 International license.

Supp Fig 2. OFT and EPM 24 hrs after adolescent DID. Across DID and sex groups, there were no differences detected in **(A)** OFT latency to enter center of arena or **(B)** OFT percent time spent in center of arena. **(C)** OFT total distance traveled was significantly influenced by DID ($F_{1,35}$ = 4.9, *p* = 0.0321) and sex ($F_{1,35}$ = 5.0, *p* = 0.0341). Secondary analyses in only alcohol-exposed subjects found that **(D)** OFT latency to enter center was significantly lower in males than in females (male: -4.5008 ± 1.5347, p = 0.0098), decreased by total alcohol consumed (ado EtOH: -0.03576 ± 0.0082, p = 0.0005), and influenced by a sex by total alcohol interaction (male x ado EtOH: 0.03393 ± 0.01395, p = 0.0271). Post hoc follow-up analyses within sex groups revealed that total alcohol consumption significantly decreased OFT latency to enter the center in females (ado EtOH: -0.2946 ± 0.0658, p = 0.0021) but not in males. **(E)** OFT percent time in center was not associated with adolescent EtOH consumption. **(F)** OFT total distance traveled was significantly increased by total adolescent EtOH consumption (ado EtOH: 8.5896 ± 3.693, p = 0.0335). Across DID and sex groups, there were no differences in **G)** EPM latency to enter an open arm, **(H)** EPM percent time spent in open arms, or **(I)** EPM percent open arm entries. In analyses examining only alcohol-treated subjects, **(J)** EPM latency to enter an open arm, **(K)** EPM percent time spent in open arms, and **(L)** EPM percent open arm entries were not significantly associated with total adolescent EtOH consumption. DID and sex did not influence **M)** EPM number of head dips or **(N)** EPM total distance traveled. In analyses of only alcohol-exposed subjects, **(O)** EPM number of head dips was not associated with total adolescent EtOH consumption. However, **(P)** EPM total distance traveled was influenced by an interaction of total adolescent EtOH consumption and sex (male x ado EtOH: 16.718 ± 6.5302, p = 0.0210). Post hoc follow-up analyses within sex groups revealed that EPM total distance traveled was significantly increased by total adolescent alcohol consumption in males (ado EtOH: 18.88 ± 5.726, p = 0.0110) but not in females. M = male; f = female; ado = adolescent. In scatter plots, a simple linear regression was used to generate lines of best fit and aid interpretation but were independent from statistical analysis. Data are shown as mean +/- SEM. *p<0.05. n=9-10/sex/treatment.

bioRxiv preprint doi: https://doi.org/10.1101/2025.06.05.658112; this version posted June 8, 2025. The copyright holder for this preprint (which was not certified by peer review) is the author/funder, who has granted bioRxiv a license to display the preprint in perpetuity. It is made available under aCC-BY 4.0 International license.

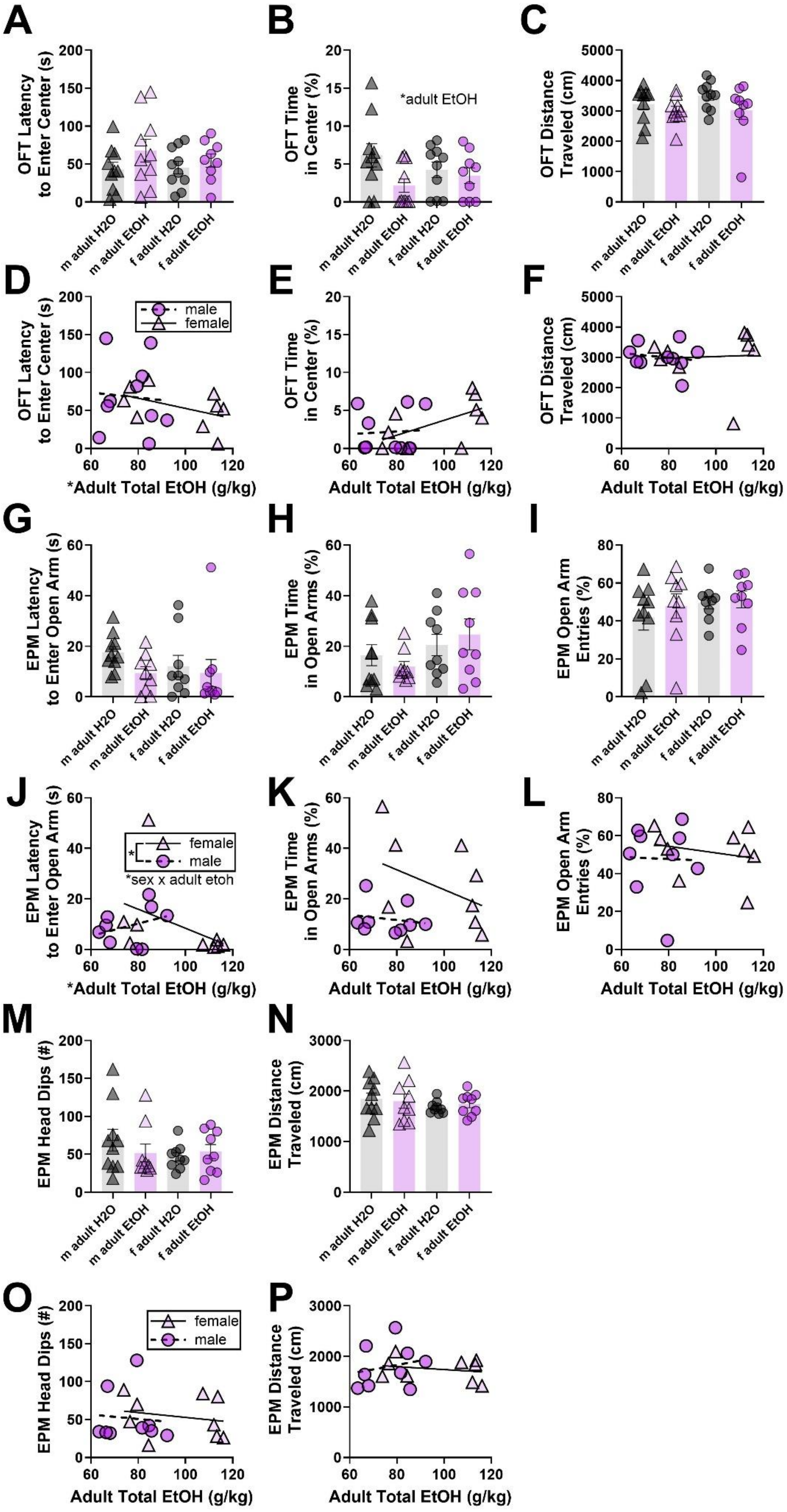

bioRxiv preprint doi: https://doi.org/10.1101/2025.06.05.658112; this version posted June 8, 2025. The copyright holder for this preprint (which was not certified by peer review) is the author/funder, who has granted bioRxiv a license to display the preprint in perpetuity. It is made available under aCC-BY 4.0 International license.

Supp Fig 3. OFT and EPM 30 days after adult DID. Across DID and sex groups, there were no differences found for **A)** OFT latency to enter center of arena. However, **(B)** OFT percent time spent in center of arena was significantly lowered after adult DID ($F_{1,35}$ = 4.3, *p* = 0.0450). **(C)** DID and sex did not influence OFT total distance traveled. In separate analyses testing effects of total alcohol consumed among only alcohol-exposed mice, **(D)** OFT latency to enter center was decreased by total alcohol consumed (adult EtOH: -0.0122 ± 0.0026, p = 0.0003). Total alcohol consumed did not significantly alter **(E)** OFT percent time in center or **(F)** OFT total distance traveled compared to adult EtOH consumption. DID and sex did not influence **G)** EPM latency to enter an open arm, **(H)** EPM percent time spent in open arms, or **(I)** EPM percent open arm entries. Analyses within alcohol-treated groups revealed that **(J)** EPM latency to enter an open arm was significantly lower in males than in females (male: -3.2651 ± 1.1546, p = 0.0134), decreased by total alcohol consumed (adult EtOH: -0.0339 ± 0.0081, p = 0.0009), and influenced by a sex by total alcohol interaction (male x adult EtOH: 0.0370 ± 0.0140, p = 0.0193). Follow-up post hoc comparisons within sex groups found that total adult alcohol consumption significantly decreased EPM latency to enter an open arm in females (adult EtOH: -0.0354 ± 0.0082, p = 0.0035) but not in males. Total adult alcohol consumption was not predictive of **(K)** EPM percent time spent in open arms and **(L)** EPM percent open arm entries. Across DID and sex groups, differences were not found in **M)** EPM number of head dips or **(N)** EPM total distance traveled. Total adult alcohol consumption was not associated with **(O)** EPM number of head dips or **(P)** EPM total distance traveled. M = male; f = female. In scatter plots, a simple linear regression was used to generate lines of best fit and aid interpretation but were independent from statistical analysis. Data are shown as mean +/- SEM. *p<0.05. n=9-10/sex/treatment.

bioRxiv preprint doi: https://doi.org/10.1101/2025.06.05.658112; this version posted June 8, 2025. The copyright holder for this preprint (which was not certified by peer review) is the author/funder, who has granted bioRxiv a license to display the preprint in perpetuity. It is made available under aCC-BY 4.0 International license.

3.4 Adolescent binge drinking led to sex-specific alterations in adult whole-brain functional connectivity and pathway-specific connectivity

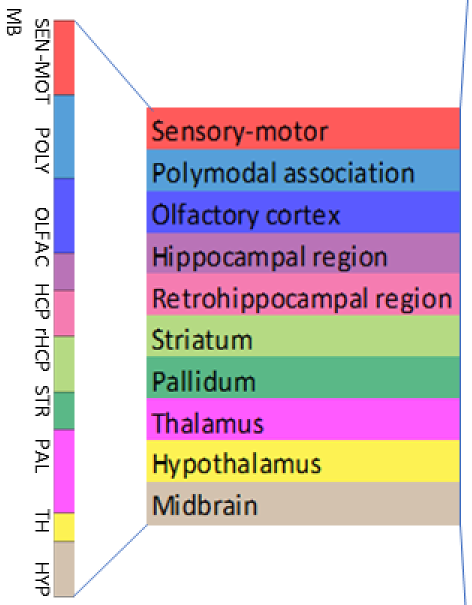

ID	Region	Region Abbv	System name	System ID
1	Gustatory areas	GU	Sensory-motor	1
2	Visceral area	VISC	Sensory-motor	1
3	Primary motor area	MOp	Sensory-motor	1
4	Secondary motor area	MOs	Sensory-motor	1
5	Primary somatosensory area	SSp	Sensory-motor	1
6	Supplemental somatosensory area	SSs	Sensory-motor	1
7	Auditory areas	AUD	Sensory-motor	1
8	visual cortex	VIS	Sensory-motor	1
9	Anterior cingulate area	ACA	Polymodal association	2
10	Prelimbic area	PL	Polymodal association	2
11	Infralimbic area	ILA	Polymodal association	2
12	Orbital area	ORB	Polymodal association	2
13	Agranular insular area	AI	Polymodal association	2
14	Retrosplenial area	RSP	Polymodal association	2
15	Parietal association areas	PTL	Polymodal association	2
16	Temporal association areas	Tev	Polymodal association	2
17	Ectorhinal area	ECT	Polymodal association	2
18	Main olfactory bulb	MOB	Olfactory cortex	3
19	Accessory olfactory bulb	AOB	Olfactory cortex	3
20	Anterior olfactory nucleus	AON	Olfactory cortex	3
21	Taenia tecta	TT	Olfactory cortex	3
22	Piriform area	PIR	Olfactory cortex	3
23	Nucleus of the lateral olfactory tract	NLOT	Olfactory cortex	3
24	Cortical amygdalar area	COA	Olfactory cortex	3
25	Postpiriform transition area	TR	Olfactory cortex	3
26	Dentate gyrus	DG	Hippocampal region	4
27	Field CA1	CA1	Hippocampal region	4
28	Field CA2	CA2	Hippocampal region	4
29	Field CA3	CA3	Hippocampal region	4
30	entorhinal area	ENT	Retrohippocampal region	5
31	presubiculum	PRE	Retrohippocampal region	5
32	postsubiculum	POST	Retrohippocampal region	5
33	parasubiculum	PAR	Retrohippocampal region	5
34	subiculum	SUB	Retrohippocampal region	5
35	Lateral septal complex	LSX	Striatum	6
36	Caudoputamen	CP	Striatum	6
37	Nucleus accumbens	ACB	Striatum	6
38	Olfactory tubercle	OT	Striatum	6
39	Fundus of striatum	FS	Striatum	6
40	Striatum-like amygdalar nuclei	sAMY	Striatum	6
41	Medial septal complex	MSC	Pallidum	7
42	Globus pallidus, external segment	GP	Pallidum	7
43	Substantia innominata	SI	Pallidum	7
44	Bed nuclei of the stria terminalis of Pallidum	BST	Pallidum	7
45	anterior hypothalamic nucleus	ATN	Thalamus	8
46	Ventral group of the dorsal thalamus	VENT	Thalamus	8
47	Lateral group of the dorsal thalamus	LAT	Thalamus	8
48	Medial group of the dorsal thalamus	MED	Thalamus	8
49	Midline group of the dorsal thalamus	MTN	Thalamus	8
50	Medial geniculate complex	MG	Thalamus	8
51	Lateral geniculate complex	LG	Thalamus	8
52	Zona incerta	ZI	Thalamus	8
53	Reticular nucleus of the thalamus	RT	Thalamus	8
54	Hypothalamic lateral zone	LZ	Hypothalamus	9
55	Periventricular zone	PVZ	Hypothalamus	9
56	Hypothalamic medial zone	MEZ	Hypothalamus	9
57	Substantia nigra	SN	Midbrain	10
58	Periaqueductal gray	PAG	Midbrain	10
59	Ventral tegmental area	VTA	Midbrain	10
60	Superior colliculus	SC	Midbrain	10
61	Midbrain reticular nucleus	MRN	Midbrain	10
62	RAmb	RAmb	Midbrain	10

bioRxiv preprint doi: https://doi.org/10.1101/2025.06.05.658112; this version posted June 8, 2025. The copyright holder for this preprint (which was not certified by peer review) is the author/funder, who has granted bioRxiv a license to display the preprint in perpetuity. It is made available under aCC-BY 4.0 International license.

Supp Fig 4. Regions of interest (ROIs) and their associated brain systems were defined based on anatomical classifications from the Allen Mouse Brain Atlas (Lein et al., 2007).

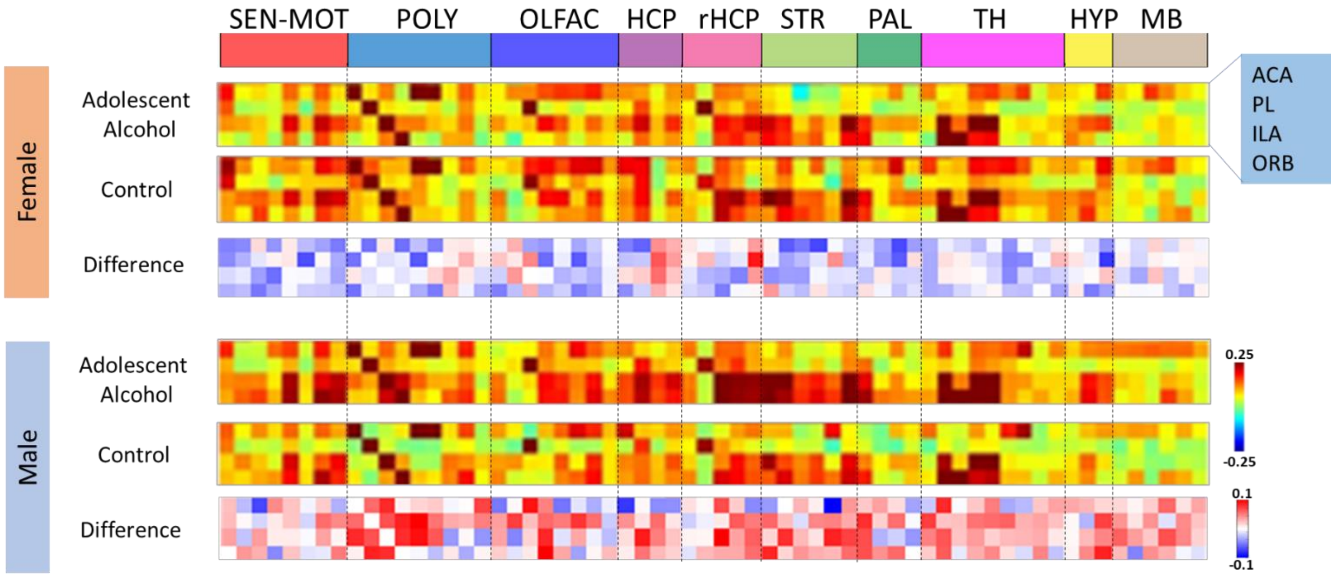

Supp Fig 5. Average functional connectivity patterns of PFC for sex- and treatment-specific groups, and sex-specific difference matrices between adolescent alcohol treatment and controls. Each matrix has 4 rows corresponding to the subregions of PFC (ACA: Anterior cingulate area, PL: Prelimbic area, ILA: Infralimbic area, and ORB: Orbital area). In each sex group, the first 2 matrices (1st, 2nd, 4th, and 5th matrices) represent the average RSFC patterns of the subregions of PFC. The color bar (with ± 0.25 range) represents the z-score values for the corresponding RSFC. The third matrices (3rd and 6th matrices) in each sex group show the difference of RSFC between alcohol-treated and control groups in their corresponding sex groups. The red-blue color bar (with ± 0.1 range) shows the z-score differences for the increased or decreased average RSFC in adolescent alcohol-treated mice in comparison to the controls, respectively.

bioRxiv preprint doi: https://doi.org/10.1101/2025.06.05.658112; this version posted June 8, 2025. The copyright holder for this preprint (which was not certified by peer review) is the author/funder, who has granted bioRxiv a license to display the preprint in perpetuity. It is made available under aCC-BY 4.0 International license.

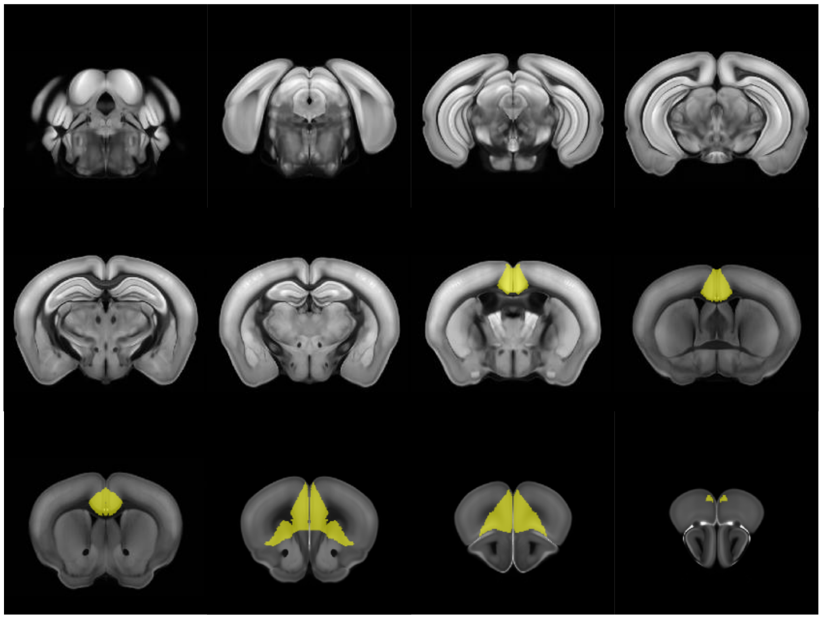

Supp Fig 6. The PFC seed in the mouse brain was defined based on anatomical classifications from the Allen Mouse Brain Atlas (Lein et al., 2007).

bioRxiv preprint doi: https://doi.org/10.1101/2025.06.05.658112; this version posted June 8, 2025. The copyright holder for this preprint (which was not certified by peer review) is the author/funder, who has granted bioRxiv a license to display the preprint in perpetuity. It is made available under aCC-BY 4.0 International license.

Supp Table 1. Significant associations between total alcohol consumed and RSFC (as displayed in Fig 4F of the main text).

ROI 1	ROI 2	Estimate
		positive relationships
Auditory areas	Ectorhinal area	0.003
Infralimbic area	anterior hypothalamic nucleus	0.003
Orbital area	Striatum-like amygdalar nuclei	0.002
Orbital area	anterior hypothalamic nucleus	0.002
Orbital area	Ventral group of the dorsal thalamus	0.002
Orbital area	Hypothalamic medial zone	0.002
Parietal association areas	Piriform area	0.002
Parietal association areas	Substantia nigra	0.003
Ectorhinal area	Postpiriform transition area	0.002
Ectorhinal area	subiculum	0.003
Ectorhinal area	Caudoputamen	0.004
Ectorhinal area	Periventricular zone	0.004
Main olfactory bulb	Bed nuclei of the stria terminalis of Pallidum	0.003
Accessory olfactory bulb	Field CA2	0.004
Accessory olfactory bulb	Periventricular zone	0.003
Accessory olfactory bulb	RAmb	0.003
Caudoputamen	anterior hypothalamic nucleus	0.003
Striatum-like amygdalar nuclei	Hypothalamic medial zone	0.003
Ventral group of the dorsal thalamus	Hypothalamic medial zone	0.003
		negative relationships
Visceral area	Ventral tegmental area	-0.003
Primary motor area	Globus pallidus, external segment	-0.002
Secondary motor area	Zona incerta	-0.003
Supplemental somatosensory area	Substantia innominata	-0.003
Supplemental somatosensory area	Lateral geniculate complex	-0.002
Auditory areas	Field CA2	-0.003
Auditory areas	Lateral septal complex	-0.004
Auditory areas	Olfactory tubercle	-0.004
Auditory areas	Globus pallidus, external segment	-0.002
visual cortex	Zona incerta	-0.003
visual cortex	Hypothalamic lateral zone	-0.003
visual cortex	Periaqueductal gray	-0.003
visual cortex	Superior colliculus	-0.003
Prelimbic area	Accessory olfactory bulb	-0.002
Prelimbic area	presubiculum	-0.002
Infralimbic area	Temporal association areas	-0.003
Agranular insular area	Lateral geniculate complex	-0.003
Temporal association areas	Lateral septal complex	-0.003
Temporal association areas	Olfactory tubercle	-0.004
Accessory olfactory bulb	Taenia tecta	-0.004

bioRxiv preprint doi: https://doi.org/10.1101/2025.06.05.658112; this version posted June 8, 2025. The copyright holder for this preprint (which was not certified by peer review) is the author/funder, who has granted bioRxiv a license to display the preprint in perpetuity. It is made available under aCC-BY 4.0 International license.

Taenia tecta	Cortical amygdalar area	-0.003
Piriform area	postsubiculum	-0.003
Piriform area	Lateral septal complex	-0.004
Piriform area	Globus pallidus, external segment	-0.003
Piriform area	Superior colliculus	-0.003
Nucleus of the lateral olfactory tract	Lateral septal complex	-0.004
Cortical amygdalar area	Field CA2	-0.003
Cortical amygdalar area	Globus pallidus, external segment	-0.002
Postpiriform transition area	Lateral septal complex	-0.003
Postpiriform transition area	Superior colliculus	-0.002
Dentate gyrus	Lateral septal complex	-0.004
Field CA2	subiculum	-0.003
Field CA2	Hypothalamic lateral zone	-0.003
Lateral septal complex	Bed nuclei of the stria terminalis of Pallidum	-0.004
Lateral septal complex	Reticular nucleus of the thalamus	-0.003
Lateral septal complex	Hypothalamic lateral zone	-0.003
Lateral septal complex	Ventral tegmental area	-0.002
Lateral septal complex	Superior colliculus	-0.004
Lateral septal complex	Midbrain reticular nucleus	-0.002
Nucleus accumbens	Reticular nucleus of the thalamus	-0.002
Nucleus accumbens	Superior colliculus	-0.004
Olfactory tubercle	Bed nuclei of the stria terminalis of Pallidum	-0.003
Olfactory tubercle	Zona incerta	-0.002
Striatum-like amygdalar nuclei	Reticular nucleus of the thalamus	-0.002
Striatum-like amygdalar nuclei	RAmb	-0.002
Medial septal complex	Ventral tegmental area	-0.002
Globus pallidus, external segment	Reticular nucleus of the thalamus	-0.003
Globus pallidus, external segment	Hypothalamic lateral zone	-0.002
Globus pallidus, external segment	Periventricular zone	-0.003
Bed nuclei of the stria terminalis of Pallidum	Lateral group of the dorsal thalamus	-0.002
Bed nuclei of the stria terminalis of Pallidum	Superior colliculus	-0.002
Lateral group of the dorsal thalamus	RAmb	-0.003
Medial group of the dorsal thalamus	Zona incerta	-0.003
Medial group of the dorsal thalamus	Ventral tegmental area	-0.002
Medial geniculate complex	Superior colliculus	-0.003
Reticular nucleus of the thalamus	Hypothalamic lateral zone	-0.002
Hypothalamic medial zone	Ventral tegmental area	-0.002
